# Supplementary material for: Host–microbiome associations in saliva predict COVID-19 severity
Source: PNAS Nexus. 2024 Mar 25;3(4):pgae126. doi: 10.1093/pnasnexus/pgae126 (PMC11010653; doi:10.1093/pnasnexus/pgae126)
Supplement: pgae126_Supplementary_Data [file pgae126_supplementary_data.docx]

**Supporting Information for**

**Host-Microbiome Associations in Saliva Predict COVID-19 Severity**

Hend Alqedari*^1,2^, Khaled Altabtbaei*^3^, Josh L. Espinoza^6,^ , Saadoun Bin-Hasan^4^, Mohammad Alghounaim^5^, Abdullah Alawady^4 ,^Abdullah Altabtabae^4^, Sarah AlJamaan^4^, Sriraman Devarajan^2^,Tahreer AlShammari^2^, Mohammed Ben Eid^4^, Michele Matsuoka^6^, Hyesun Jang^6^, Christopher L. Dupont^6^, Marcelo Freire^6,7^

**Affiliations**

^1^Department of Oral Health Policy and Epidemiology, Harvard School of Dental Medicine, Boston, MA, 02115, USA; Dasman Diabetes Institute, Kuwait

^2^Dasman Diabetes Institute, 1180, Dasman, Kuwait

^3^School of Dentistry, Faculty of Medicine and Dentistry. University of Alberta. Edmonton AB, T6G 2L7, Canada

^4^Department of Pediatrics, Farwaniyah Hospital, Ministry of Health, Kuwait

^5^Department of Pediatrics, Amiri Hospital, Ministry of Health, Kuwait

^6^Department of Genomic Medicine and Infectious Diseases, J. Craig Venter Institute, La Jolla, CA 92037, USA

^7^Division of Infectious Diseases and Global Public Health Department of Medicine, University of California San Diego, La Jolla, CA, USA

**Corresponding Author***

Marcelo Freire, D.D.S., Ph.D., D.Med.Sc.

Associate Professor

Genomic Medicine and Infectious Diseases

J. Craig Venter Institute

4120 Capricorn Lane, La Jolla, CA 92037, USA

Phone: 858-200-1846

Fax: 858-200-1880

**Email:**  [mfreire@jcvi.org](mailto:mfreire@jcvi.org)

**This PDF file includes:**

Figures S1 to S9

Tables S1 to S5

**Supplementary Figure S1**

A


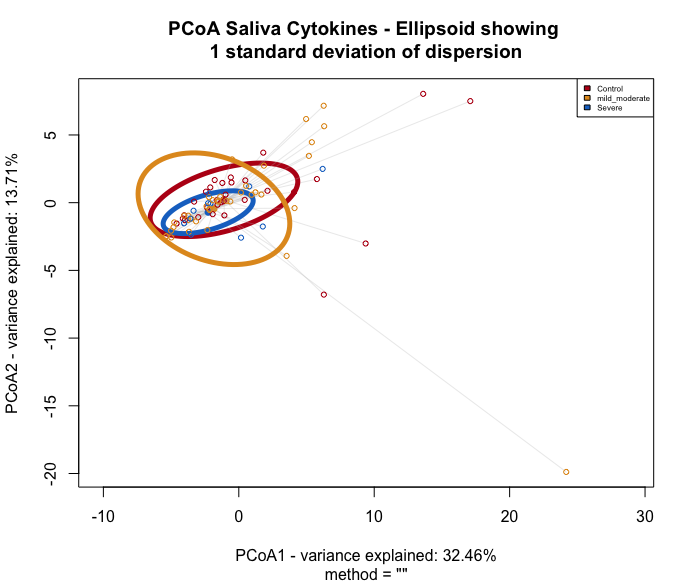


B


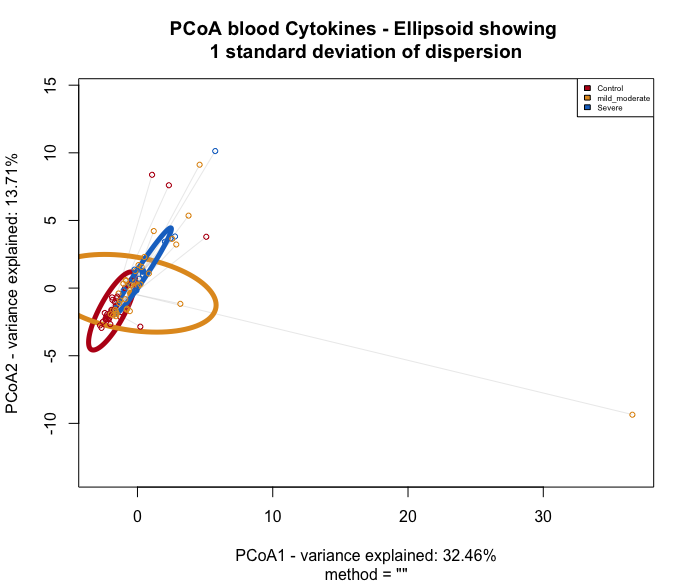


**Supplementary Figure S1.** PCoA analysis of cytokines of salivary (A) and plasma (B) microbiomes between the three groups. The cytokines were z-score standardized then graphed using Euclidean distances. The ellipsoids are mapped to show 1 standard deviation of dispersion. There was no statistically significant difference between them (p>0.05, ADONIS).

**Supplementary Figure S2**


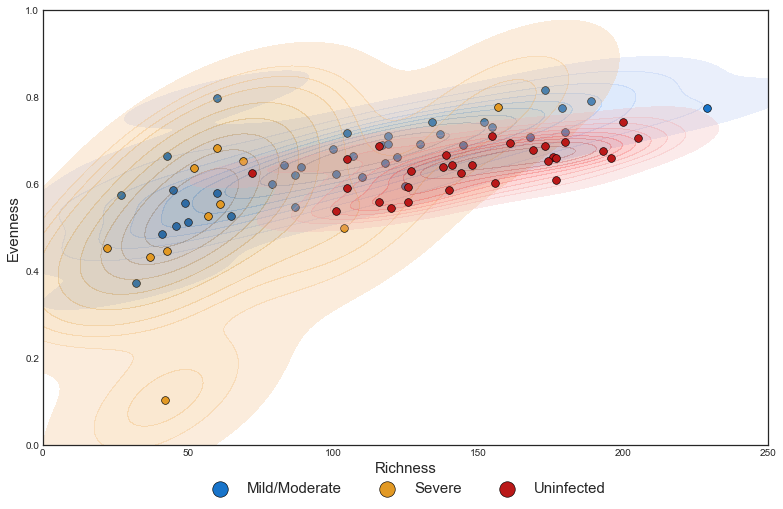


**Supplementary Figure S2 –** Scatterplots of Salivary microbiome richness (x-axis) vs. Pielou's evenness index (y-axis). Each sample is represented by a dot, and colored based on their disease status (uninfected/control – red, mild/moderate symptoms – blue, severe – yellow). Heatmap superimposed on the scatterplot to demonstrate density function.

**Supplementary Figure S3**


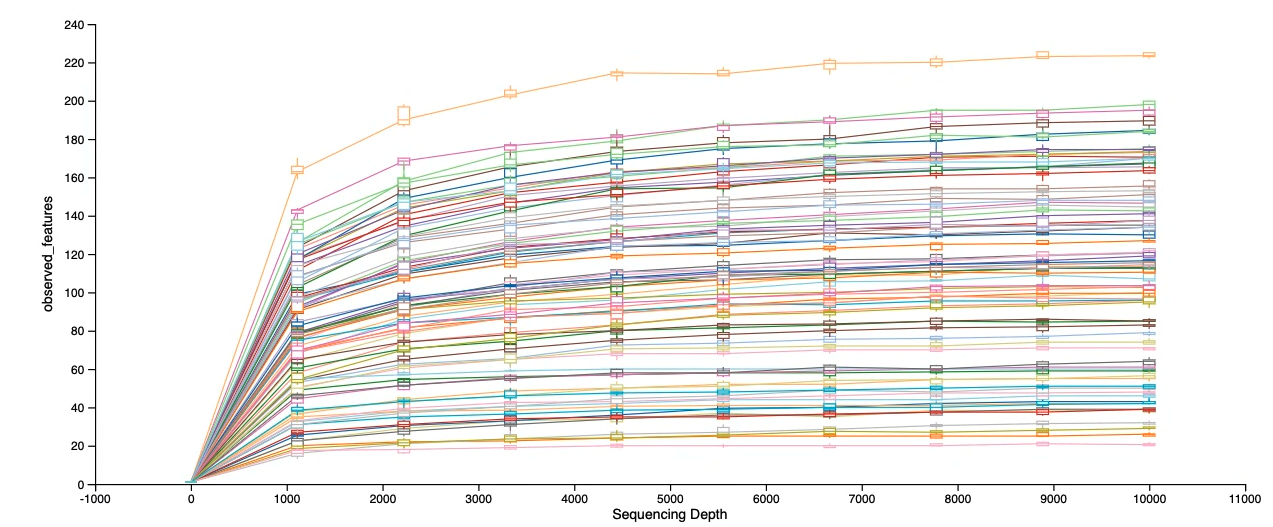


**Supplementary Figure S3.**  Alpha rarefaction curves – showing “observed features” curves. Flattening of the curve demonstrates that we have achieved adequate sequencing depth for sequences.

**Supplementary Figure S4**

**
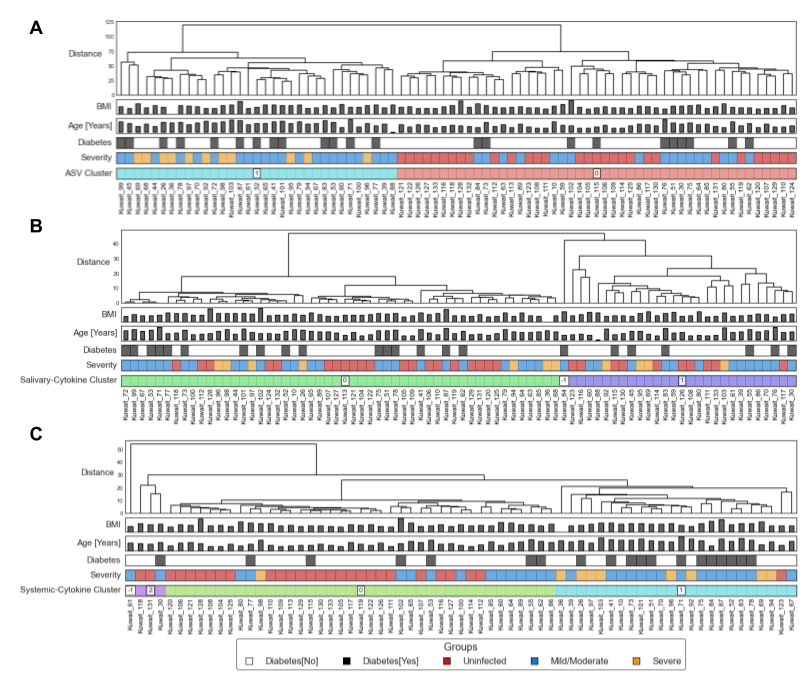
**

#### **Supplementary Figure S4.** Unsupervised clustering of saliva microbiome and cytokines. (A) Clustering using ASV abundances. (80 samples, 463 ASVs). Bottom row showing the first tree bifurcation, which has clustered most COVID-19 positive samples together. (B) Clustering using salivary cytokine abundances. (80 samples, 65 cytokines). (C) Clustering using systemic cytokine abundances. (80 samples, 65 cytokines).

**Supplementary Figure S5**


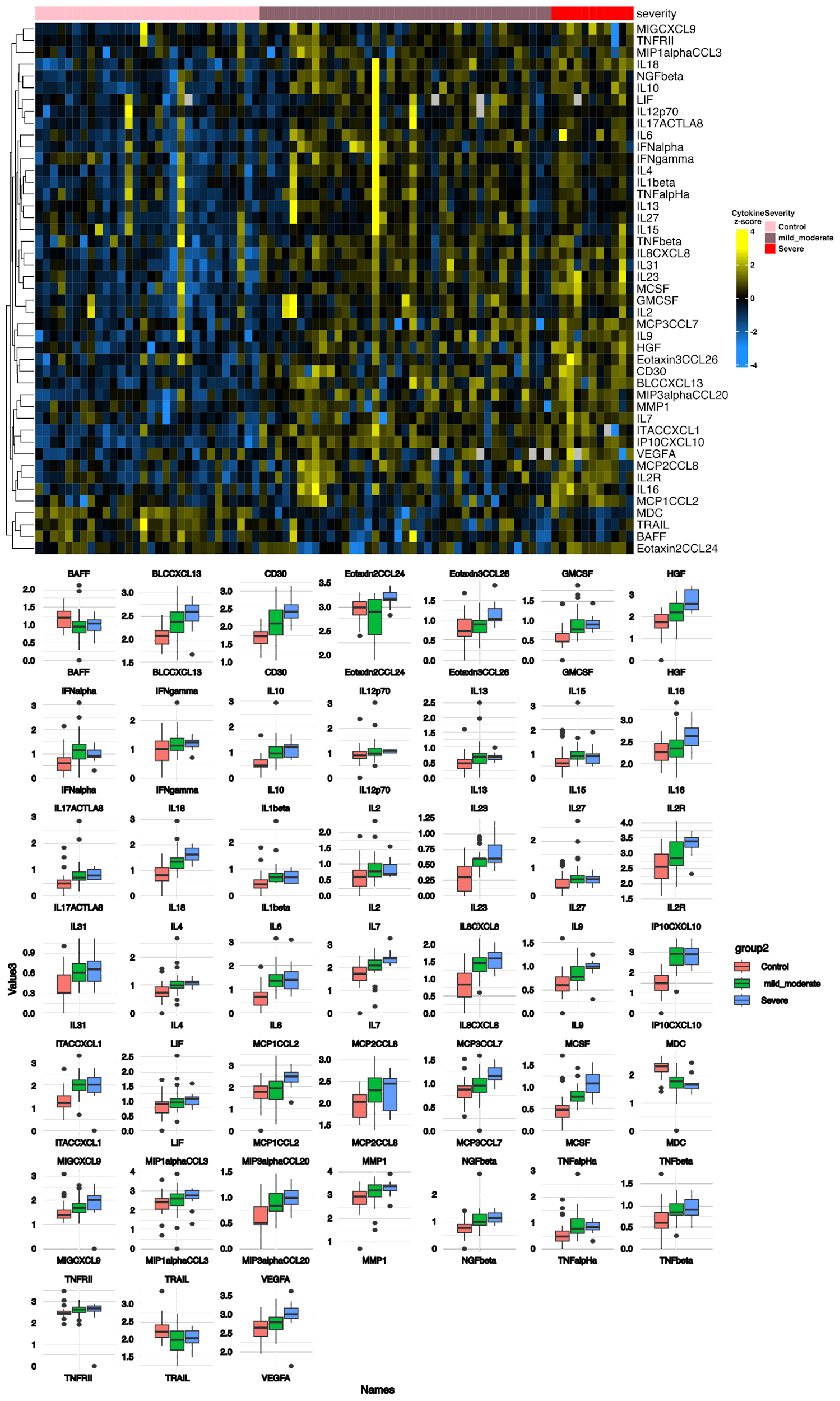


**Supplementary Figure S5.** Boxplot of representative blood cytokines (p<0.05, Kruskal-Wallis test). All values were log10 transformed before graphing. Whiskers demonstrate IQR.

**Supplementary Figure S6**


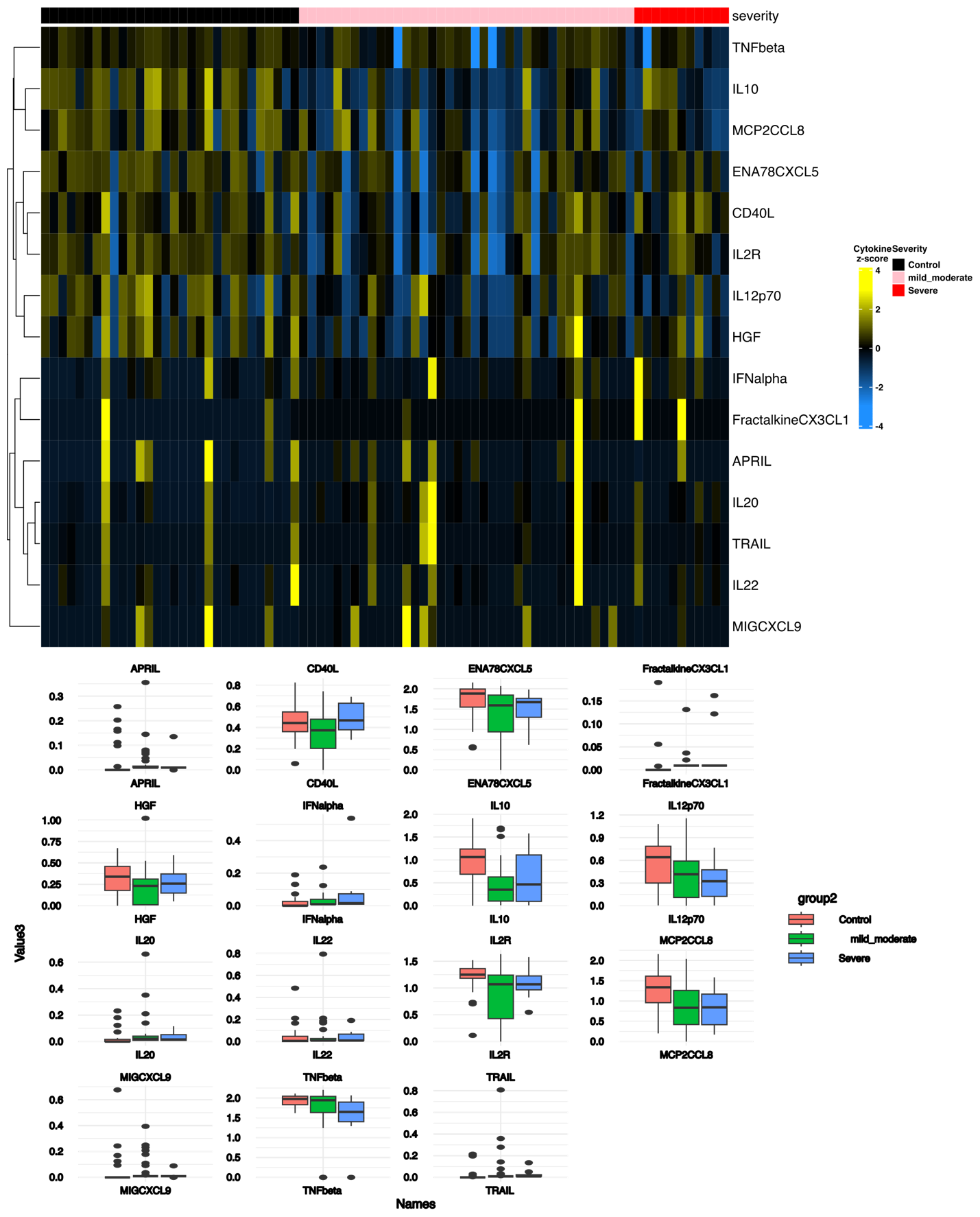


**Supplementary Figure S6.** Boxplot of representative saliva cytokines. (p<0.05, Kruskal-Wallis test). All values were log10 transformed before graphing. Whiskers demonstrate IQR.

**Supplementary Figure S7**
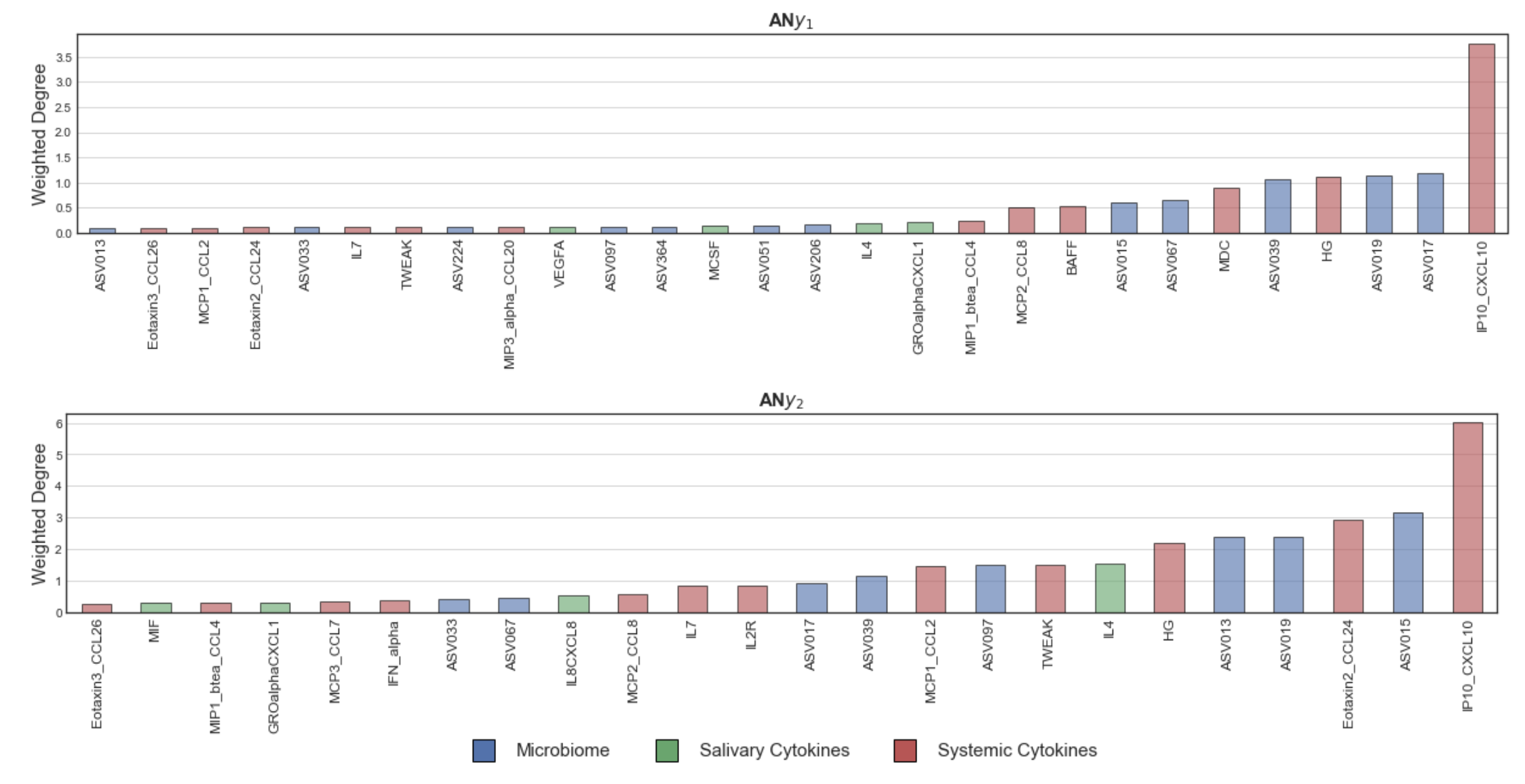


**Supplementary Figure S7**. Weighted degree of nodes in aggregate networks for sub-models y1 and y2. Node the high degree of connectivity of IP10/CXCL10 in y1 – the sum of degrees of ASV017 (Veillonella sp.), ASV019 (Pasteurellaceae sp.), systemic HG, and ASV039 (Corynebacterium matruchotii) s is 4.55 while CXCL10/IP-10 alone is 3.77.

**Supplementary Figure S8**

**
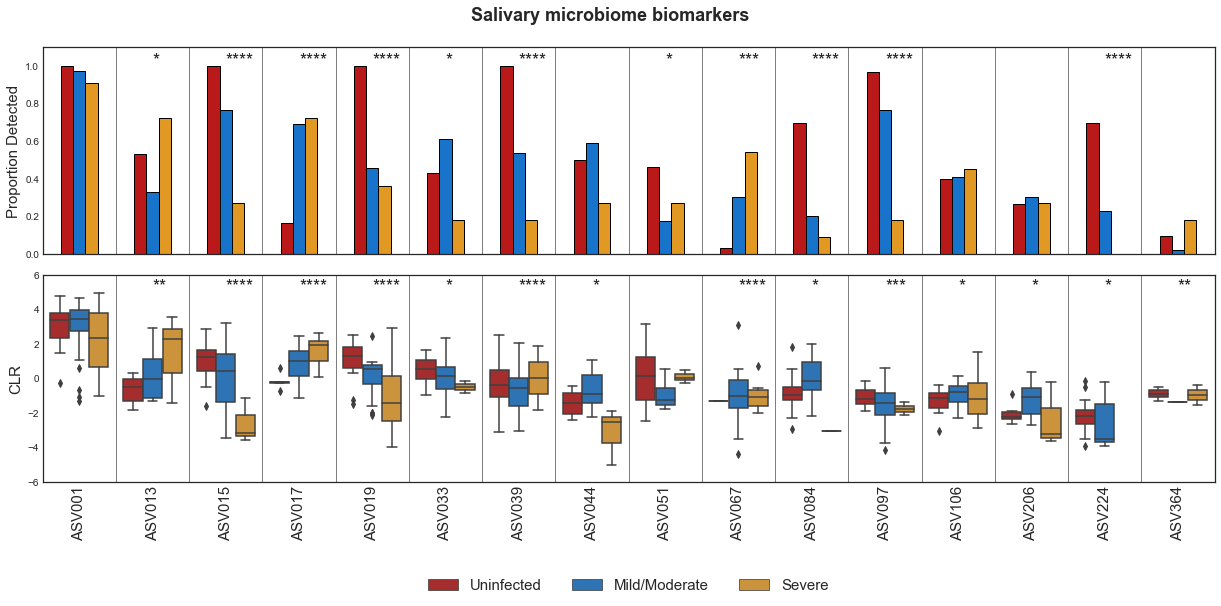
**

**Supplementary Figure S8.** Statistical significance for salivary microbiome biomarkers in the context of (top) detected vs. not detected using a Fisher’s Exact test and (bottom) CLR values using a Kruskal-Wallis H-test. FDR < 0.05 *, FDR < 0.01 **, FDR < 0.001 ***, and FDR < 0.0001 ****.

**Supplementary Figure S9**

**Supplementary Figure S9.** Average Net MFI data for the cytokines were normalized then log10 transformed. Baseline cutoff was placed to 0.

**
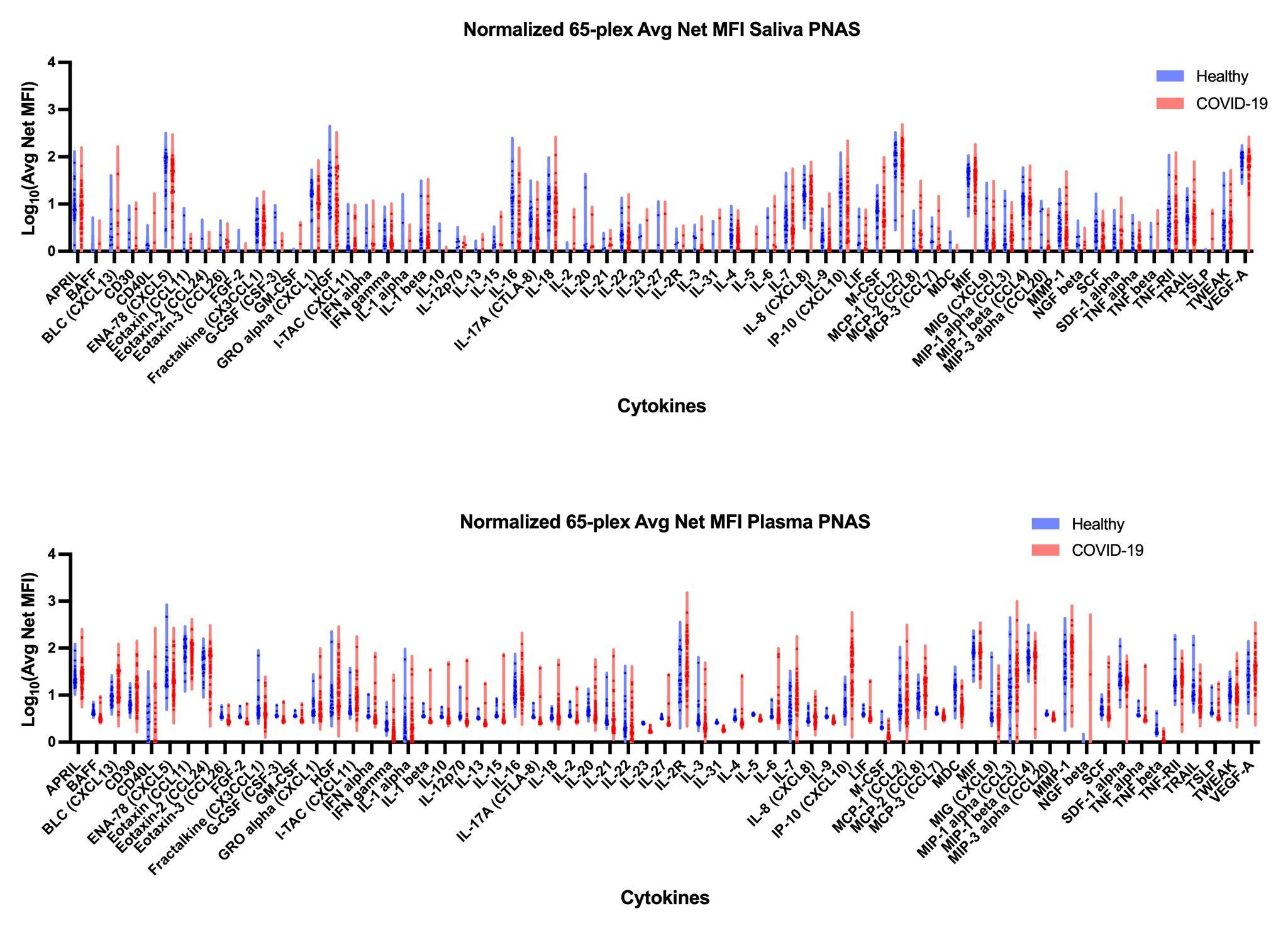
**

**Supplementary tables**

#### **Supplementary Table S1.** Biomarker descriptors for abundance-based paradigms with respect to sub-model

| **Sub-model** | **Modality** | **Features** |
| --- | --- | --- |
| **y1** | **Microbiome** | ASV017, ASV019, ASV033, ASV067, ASV001, ASV084, ASV039, ASV224, ASV051, ASV206 |
|  | **Salivary** | IL16, ENA78CXCL5, VEGFA, IL17ACTLA8, IL5, TWEAK, FGF2, FractalkineCX3CL1, HGF, GROalphaCXCL1, MCP2CCL8, NGFbeta, MIP1alphaCCL3, MCP1CCL2, MIF, IL27, TNFalpha, MMP1, IL4, MCSF, TNFRII, MIGCXCL9, IP10CXCL10, IL1beta, GCSFCSF3, IL10 |
|  | **Systemic** | MDC, IP10_CXCL10, HG, MCP2_CCL8, MIP1_btea_CCL4, MIP3_alpha_CCL20 |
| **y2** | **Microbiome** | ASV013, ASV015, ASV044, ASV106, ASV364, ASV097 |
|  | **Salivary** | IL4, FGF2, VEGFA, IP10CXCL10, IL16, MMP1, TNFalpha, MIF, IL10, IL8CXCL8, IL6, IL5 |
|  | **Systemic** | Eotaxin2_CCL24, MIG_CXCL9, MCSF, MCP3_CCL7, TWEAK, HG, MIP1_btea_CCL4, IL7, MCP1_CCL2, BAFF, Eotaxin3_CCL26, IL2R, IFN_alpha, APRIL, IL22 |

**Supplementary Table 2**. Taxonomic lineage for salivary microbiome biomarkers.

| **ASV_ID** | **Phylum** | **Class** | **Order** | **Family** | **Genus** | **Species** |
| --- | --- | --- | --- | --- | --- | --- |
| **ASV001** | Firmicutes | Negativicutes | Veillonellales-Selenomonadales | Veillonellaceae | Veillonella |  |
| **ASV013** | Firmicutes | Negativicutes | Veillonellales-Selenomonadales | Veillonellaceae | Veillonella |  |
| **ASV015** | Firmicutes | Bacilli | Staphylococcales | Gemellaceae | Gemella |  |
| **ASV017** | Firmicutes | Negativicutes | Veillonellales-Selenomonadales | Veillonellaceae | Veillonella |  |
| **ASV019** | Proteobacteria | Gammaproteobacteria | Pasteurellales | Pasteurellaceae |  |  |
| **ASV033** | Actinobacteriota | Actinobacteria | Micrococcales | Micrococcaceae | Rothia |  |
| **ASV039** | Actinobacteriota | Actinobacteria | Corynebacteriales | Corynebacteriaceae | Corynebacterium | Corynebacterium_matruchotii |
| **ASV044** | Bacteroidota | Bacteroidia | Flavobacteriales | Flavobacteriaceae | Capnocytophaga | Capnocytophaga_granulosa |
| **ASV051** | Firmicutes | Negativicutes | Veillonellales-Selenomonadales | Veillonellaceae | Veillonella | uncultured_methanogenic |
| **ASV067** | Cyanobacteria | Cyanobacteriia | Chloroplast | Chloroplast | Chloroplast |  |
| **ASV084** | Bacteroidota | Bacteroidia | Bacteroidales | Prevotellaceae | Alloprevotella | Alloprevotella_rava |
| **ASV097** | Firmicutes | Bacilli | Erysipelotrichales | Erysipelotrichaceae | Solobacterium |  |
| **ASV106** | Fusobacteriota | Fusobacteriia | Fusobacteriales | Leptotrichiaceae | Leptotrichia | Leptotrichia_wadei |
| **ASV206** | Actinobacteriota | Actinobacteria | Bifidobacteriales | Bifidobacteriaceae | Alloscardovia | Alloscardovia_omnicolens |
| **ASV224** | Bacteroidota | Bacteroidia | Bacteroidales | Rikenellaceae | Rikenellaceae_RC9_gut_group | Bacteroidales_oral |
| **ASV364** | Firmicutes | Negativicutes | Veillonellales-Selenomonadales | Selenomonadaceae |  |  |

**Supplementary Table 3**. Periodontal condition between COVID-19 positive and control.

|  | COVID-19 negative  n = 30 (%) | COVID-19 positive  n = 50 (%) | Total (%) | P value |
| --- | --- | --- | --- | --- |
| No periodontal disease | 21 (70%) | 25 (50%) | 46 (57.5) | 0.08 |
| Periodontal disease | 9 (30%) | 25 (50%) | 34 |  |

We classified a subject as having periodontal disease if they responded positively to at least one of the following criteria: the presence of existing bone loss, loose teeth, or gum inflammation.

**Supplementary Table 4**. Periodontal condition between COVID-19 severity status.

| Periodontal condition | Control  n=30 (%) | Mild/moderate COVID-19 symptoms  n=39 (%) | Severe COVID-19 symptoms  n = 11 (%) | Total (%) | P value |
| --- | --- | --- | --- | --- | --- |
| No periodontal disease | 21 (70) | 19 (48.7) | 6 (54.5) | 46 (57.5) | 0.2 |
| Periodontal disease | 9 (30) | 20 (51.3) | 5 (45.5) | 34 (42.5) |  |

We classified a subject as having periodontal disease if they responded positively to at least one of the following criteria: the presence of existing bone loss, loose teeth, or gum inflammation.

**Supplementary Table 5**. List and range of detection for cytokines.

| **SALIVA** | | | **PLASMA** | | |
| --- | --- | --- | --- | --- | --- |
| **Cytokine** | **Upper Limit** | **Lower Limit** | **Cytokine** | **Upper Limit** | **Lower Limit** |
| APRIL | 180500 | 44.07 | APRIL | 757800 | 185 |
| BAFF | 23300 | 5.69 | BAFF | 9600 | 2.34 |
| BLC (CXCL13) | 45200 | 11.04 | BLC (CXCL13) | 62100 | 15 |
| CD30 | 31800 | 7.76 | CD30 | 35000 | 8.54 |
| ENA-78 (CXCL5) | 15100 | 3.69 | ENA-78 (CXCL5) | 32600 | 7.96 |
| Eotaxin-2 (CCL24) | 16600 | 4.05 | Eotaxin-2 (CCL24) | 22200 | 5.42 |
| Eotaxin-3 (CCL26) | 6000 | 1.47 | Eotaxin-3 (CCL26) | 1638 | 1.6 |
| FGF-2 | 18600 | 4.54 | FGF-2 | 41800 | 10 |
| Fractalkine (CX3CL1) | 10600 | 2.59 | Fractalkine (CX3CL1) | 2900 | 2.83 |
| I-TAC (CXCL1) | 39900 | 9.74 | I-TAC (CXCL1) | 12425 | 12 |
| IL-16 | 55200 | 13.48 | IL-16 | 64300 | 16 |
| IL-20 | 29000 | 7.08 | IL-20 | 14200 | 14 |
| IL-2R | 333300 | 81.37 | IL-2R | 411200 | 100 |
| MCP-2 (CCL8) | 3400 | 0.83 | MCP-2 (CCL8) | 1013 | 0.99 |
| MCP-3 (CCL7) | 18900 | 4.62 | MCP-3 (CCL7) | 18000 | 4.39 |
| MDC | 75200 | 18.36 | MDC | 79100 | 19 |
| MIF | 2450 | 0.6 | MIF | 925 | 0.9 |
| MIG (CXCL9) | 35100 | 8.57 | MIG (CXCL9) | 10225 | 9.99 |
| TNF-RII | 14700 | 3.59 | TNF-RII | 12000 | 2.93 |
| TRAIL | 46800 | 11.43 | TRAIL | 13900 | 3.39 |
| TSLP | 27500 | 6.71 | TSLP | 4000 | 3.91 |
| TWEAK | 368300 | 89.92 | TWEAK | 564300 | 138 |
| CD40L | 19100 | 4.66 | CD40L | 50900 | 12 |
| Eotaxin (CCL11) | 6550 | 1.6 | Eotaxin (CCL11) | 2038 | 1.99 |
| G-CSF (CSF-3) | 40400 | 9.86 | G-CSF (CSF-3) | 14375 | 14 |
| GM-CSF | 53600 | 13.09 | GM-CSF | 55700 | 14 |
| GRO alpha (CXCL1) | 10000 | 2.44 | GRO alpha (CXCL1) | 3625 | 3.54 |
| HGF | 13400 | 3.27 | HGF | 27000 | 6.59 |
| IFN alpha | 19700 | 4.81 | IFN alpha | 36300 | 8.86 |
| IFN gamma | 43700 | 10.67 | IFN gamma | 43800 | 11 |
| IL-1 alpha | 6500 | 1.59 | IL-1 alpha | 7750 | 1.89 |
| IL-1 beta | 14100 | 3.44 | IL-1 beta | 28600 | 7.23 |
| IL-10 | 9850 | 2.4 | IL-10 | 17000 | 4.15 |
| IL-12p70 | 31300 | 7.64 | IL-12p70 | 29600 | 7.23 |
| IL-13 | 14300 | 3.49 | IL-13 | 19300 | 4.71 |
| IL-15 | 15800 | 3.86 | IL-15 | 12800 | 3.13 |
| IL-17A (CTLA-8) | 95600 | 23.34 | IL-17A (CTLA-8) | 15500 | 3.78 |
| IL-18 | 28700 | 7.01 | IL-18 | 39900 | 9.74 |
| IL-2 | 79100 | 19.31 | IL-2 | 40900 | 9.99 |
| IL-21 | 28000 | 6.83 | IL-21 | 41500 | 10 |
| IL-22 | 81700 | 19.95 | IL-22 | 71700 | 18 |
| IL-23 | 66300 | 16.19 | IL-23 | 62200 | 15 |
| IL-27 | 61200 | 14.94 | IL-27 | 42200 | 10 |
| IL-3 | 94700 | 23.12 | IL-3 | 130300 | 32 |
| IL-31 | 50500 | 12.33 | IL-31 | 65800 | 16 |
| IL-4 | 79500 | 19.41 | IL-4 | 53100 | 13 |
| IL-5 | 27700 | 6.76 | IL-5 | 41900 | 10 |
| IL-6 | 21300 | 5.2 | IL-6 | 51500 | 13 |
| IL-7 | 2100 | 0.51 | IL-7 | 3950 | 0.96 |
| IL-8 (CXCL8) | 8150 | 1.99 | IL-8 (CXCL8) | 11100 | 2.71 |
| IL-9 | 43700 | 10.67 | IL-9 | 30500 | 7.45 |
| IP-10 (CXCL10) | 10300 | 2.52 | IP-10 (CXCL10) | 2288 | 2.23 |
| LIF | 18500 | 4.52 | LIF | 19000 | 4.64 |
| M-CSF | 259600 | 63.38 | M-CSF | 66100 | 16 |
| MCP-1 (CCL2) | 15100 | 3.69 | MCP-1 (CCL2) | 2625 | 2.56 |
| MIP-1 alpha (CCL3) | 10000 | 2.44 | MIP-1 alpha (CCL3) | 3175 | 3.1 |
| MIP-1 beta (CCL4) | 12800 | 3.13 | MIP-1 beta (CCL4) | 9275 | 9.06 |
| MIP-3 alpha (CCL20) | 67600 | 16.5 | MIP-3 alpha (CCL20) | 46200 | 11 |
| MMP-1 | 19400 | 4.74 | MMP-1 | 17100 | 4.17 |
| NGF beta | 16500 | 4.03 | NGF beta | 24200 | 5.91 |
| SCF | 9750 | 2.38 | SCF | 18600 | 4.54 |
| SDF-1 alpha | 158900 | 38.8 | SDF-1 alpha | 79450 | 78 |
| TNF alpha | 20100 | 4.91 | TNF alpha | 15000 | 15 |
| TNF beta | 24900 | 6.08 | TNF beta | 28900 | 7.06 |
| VEGF-A | 21500 | 5.25 | VEGF-A | 24700 | 6.03 |
